# Clinical and radiological midterm outcome after treatment of pilonoidal fracture dislocations of the proximal interphalangeal joint with a parabolic dynamic external fixator

**DOI:** 10.1007/s00402-019-03275-8

**Published:** 2019-09-05

**Authors:** T. Kastenberger, P. Kaiser, M. Keller, G. Schmidle, M. Gabl, Rohit Arora

**Affiliations:** 1 Department of Trauma Surgery, Medical University of Innsbruck, Anichstr. 35, 6020 Innsbruck, Austria; 2 Department of Orthopedics and Traumatology, Kantonsspital Baselland, Liestal, Switzerland

**Keywords:** Dynamic, External fixation, Fracture, Proximal interphalangeal joint, Dislocation, Treatment

## Abstract

**Introduction:**

Several methods treating proximal interphalangeal joint (PIP) fracture dislocations have been established providing early joint mobilization. The aim of this study was to evaluate the clinical and radiological outcome of unstable fracture dislocations of the PIP treated with a parabolic dynamic external fixator consisting of two Kirschner (K)-wires.

**Materials and methods:**

Twenty-one patients who sustained a pilonoidal fracture of the PIP joint and were treated with a dynamic external fixator were evaluated retrospectively. The active range of motion, pain level, DASH score, Buck Gramcko Score, and the patient’s satisfaction and acceptance were assessed. X-ray images were evaluated for bone healing, joint alignment, and signs of osteoarthritis.

**Results:**

Mean PIP joint range of motion was 76°. Patients showed very mild discomfort (mean 0.7), high patient satisfaction (mean 1.9), and a moderate acceptance (mean 2.7). The mean DASH score was 11.6 and the Buck Gramcko score 13. All patients showed bone healing. One patient suffered from a recurrent dislocation, and another a subluxation of the PIP joint while wearing the fixator. Both joints could be corrected by modifying the fixator under image intensifier. Twenty patients (95%) showed a concentric and stable aligned joint. Three patients showed an osteoarthritis stage 0, five stage 1, nine stage 2, three stage 3, and one stage 4 according to the Kellgran–Lawrence Score.

**Conclusion:**

The use of a parabolic dynamic external fixator constructed from two K-wires restores joint alignment and stability in unstable pilonoidal PIP joint disclocation fractures. It allows immediate PIP joint mobilization to avoid adhesions. Modifications of the radius of the parabolic construct within cases of postoperative malalignment, without anesthesia, can restore joint axis and malalignment. This fixator is a cost-effective alternative, showing a good clinical outcome.

## Introduction

Excessive axial load to the finger can cause a fracture dislocation of the proximal interaphalangeal (PIP) joint. The middle phalanx is impacted at the head of the proximal phalanx, fracturing the articular surface of the base of the middle phalanx. A palmar, a dorsal, and/or an impressed central fracture fragment can occur depending on the load transition. In cases of fracture dislocation or joint subluxation, surgical treatment seems mandatory for a satisfactory result.

Different surgical treatment options have been described including extension block pinning, open reduction and internal fixation (ORIF) with screws or plating, percutaneous Kirschner (K)-wire pinning after closed or mini-open reduction, and various different external traction fixation systems including K-wires, rubber bands, and springs [1–3].

Because open reduction and fixation is difficult and may lead to fragment necrosis, closed reduction including traction systems is favored. These systems allow early mobilization, whereas the immobilization of the PIP joint results in stiffness with a reduced range of motion due to periarticular scarring [4]. The use of an external traction fixator is based on reduction through traction of the fracture components and ligamentotaxis within the PIP joint to correct any subluxation of the joint [5–10]. Good clinical and radiological results can be expected if anatomical alignment and congruity of the joint can be restored together with sufficient stability for early joint mobilization. There are limited data in the literature regarding the outcome of an external traction fixation system which uses K-wires without rubber bands or springs.

The purpose of this retrospective study was to evaluate the clinical and radiological midterm outcome of the parabolic external dynamic fixation system described by Syed et al. [7] used for pilonoidal PIP joint fracture dislocation injuries.

## Patients and methods

All patients with a fracture dislocation of the PIP joint who were treated with a dynamic external fixator at our institution between 2005 and 2015 were enrolled in this study. Inclusion criteria include a multifragmented and displaced base fracture of the middle phalanx with an articular impression including more than 40% of the joint surface, and/or a dorsal subluxation of the middle phalanx and surgical treatment at our institution.

Twenty-five patients met the inclusion criteria; however, only 21 patients (6 male, 15 female, mean age 37.7 years; range 15–73 years) were available for the final follow-up. All patients were treated with the dynamic external fixator described by Syed et al. [7].

### Surgical technique

Between 2005 and 2014, the surgery was performed under general or axial block anesthesia. From 2014 onwards, the WALANT technique was used for anesthesia. The advantage of the latter method is that the patient can actively move the PIP joint during surgery. Therefore, any restrictions of active motion due to joint incongruency can be addressed immediately intraoperatively.

**Fig. 1:**
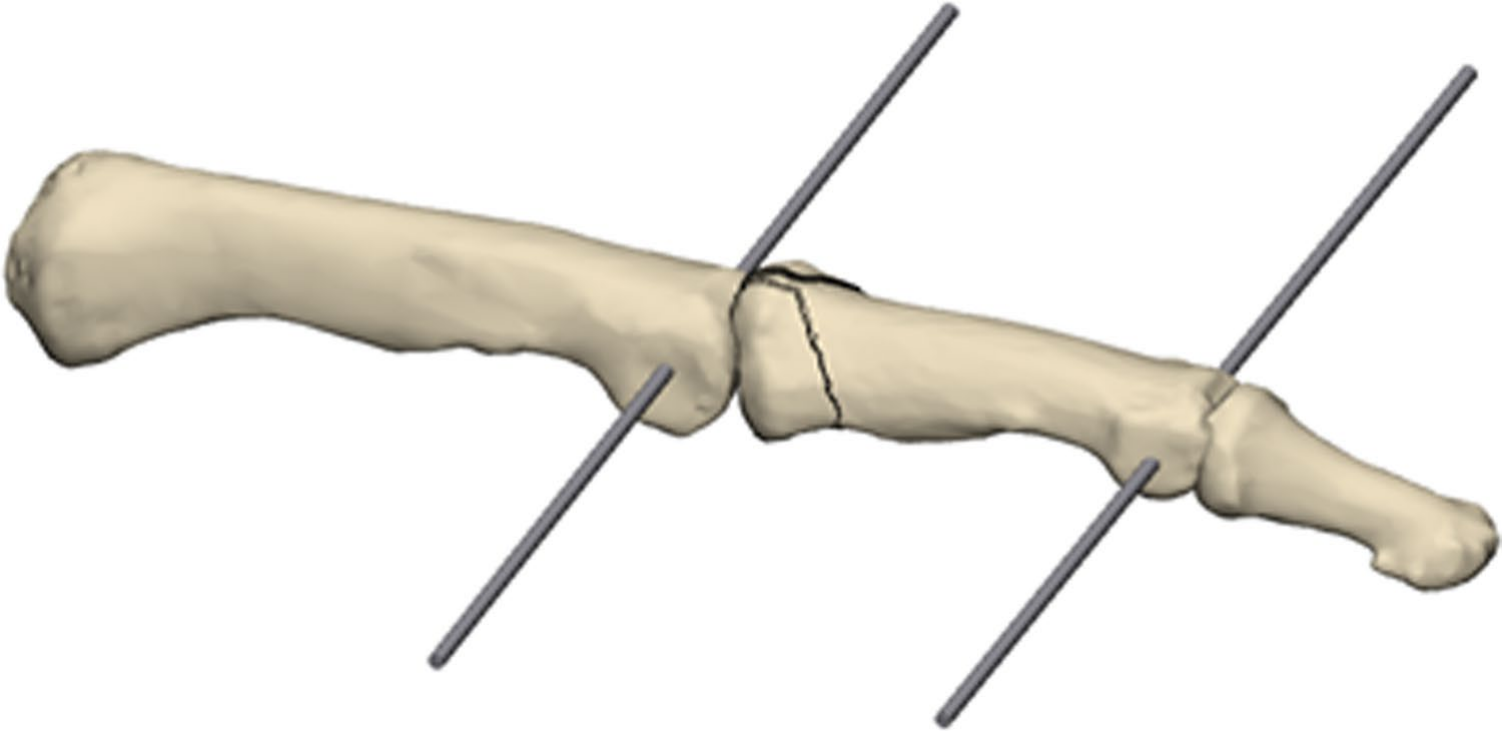
Placement of the two K-wires

1.2 mm K-wires were used for the ring and small finger, while 1.4 mm K-wires were used for the index and middle finger. The first K-wire was inserted into the head of the proximal phalanx and the second K-wire was inserted into the head of the middle phalanx, both perpendicular to the longitudinal axis in the coronal plane and parallel to the PIP joint axis (Fig. 1).

**Fig. 2:**
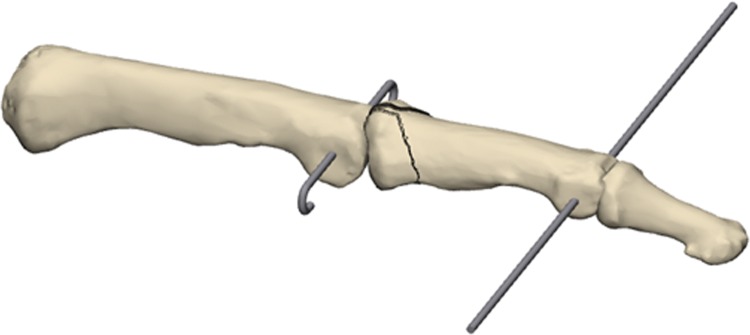
Bending of the proximal K-wire

In the lateral projection, the K-wires have to be placed in the center of the trochlea of the proximal and middle phalanx according to the rotational center, avoiding the collateral ligaments. The free ends of the proximal K-wire were bent 90°, to form a hook, and then shortened (Fig. 2).

**Fig. 3:**
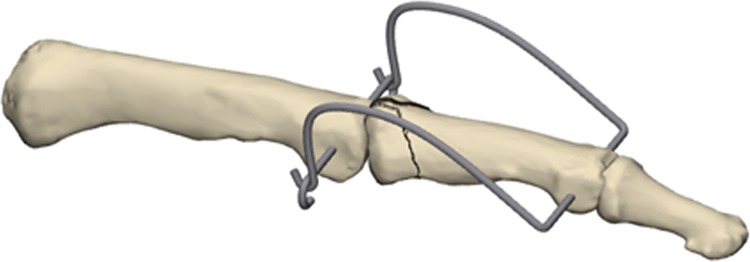
Bending of the distal K-wire

The free ends of the distal K-wire were consecutively bent in the sagittal plane in a dorsoproximal direction, forming a Fibonacci-curve shape using two mini-pliers. Special attention was paid to the distance between the bending point and the finger to not compromise the skin in cases of a postoperative swelling. Finally, the ends of the distal K-wires were formed into a hook, shortened, and hooked to the proximal K-wire (Fig. 3).

**Fig. 4:**
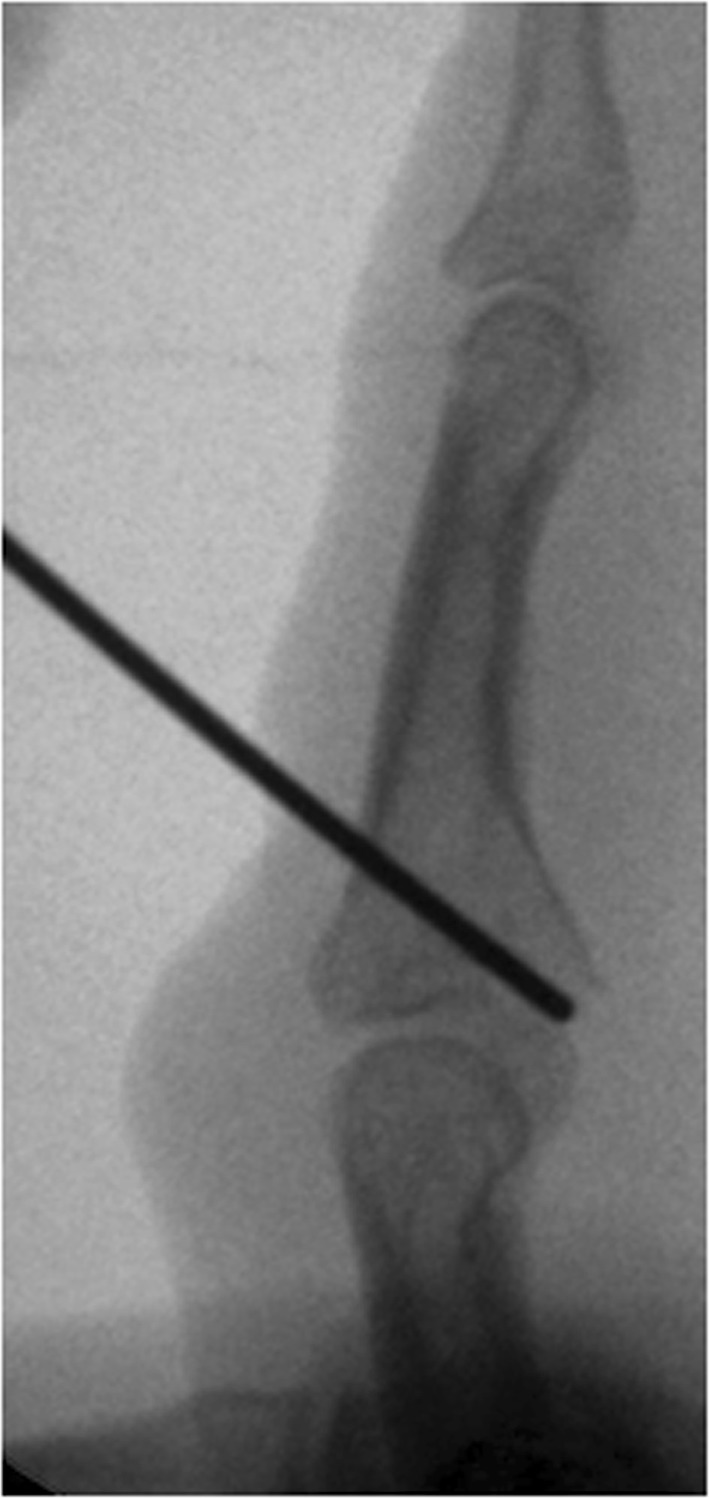
Manipulation of a depressed fragment with a K-wire through the fraction gap

If the joint impression at the base of the middle phalanx could not be corrected by a closed reduction maneuver, a 1.0 mm K-wire was inserted dorsally inside the fracture gap and used as a joystick to elevate the depressed fragments (Fig. 4).

**Fig. 5:**
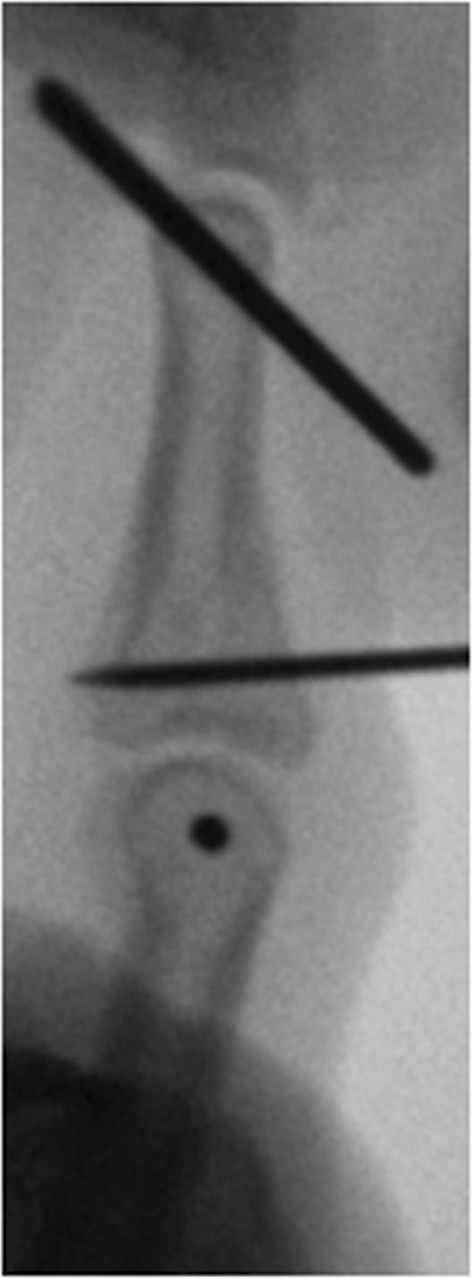
Indirect fixation of a palmar fragment with a K-wire

If necessary, large palmar fragment can be fixed indirectly by one or two K-wires, inserted dorsally, parallel to the joint surface (Fig. 5).

**Fig. 6:**
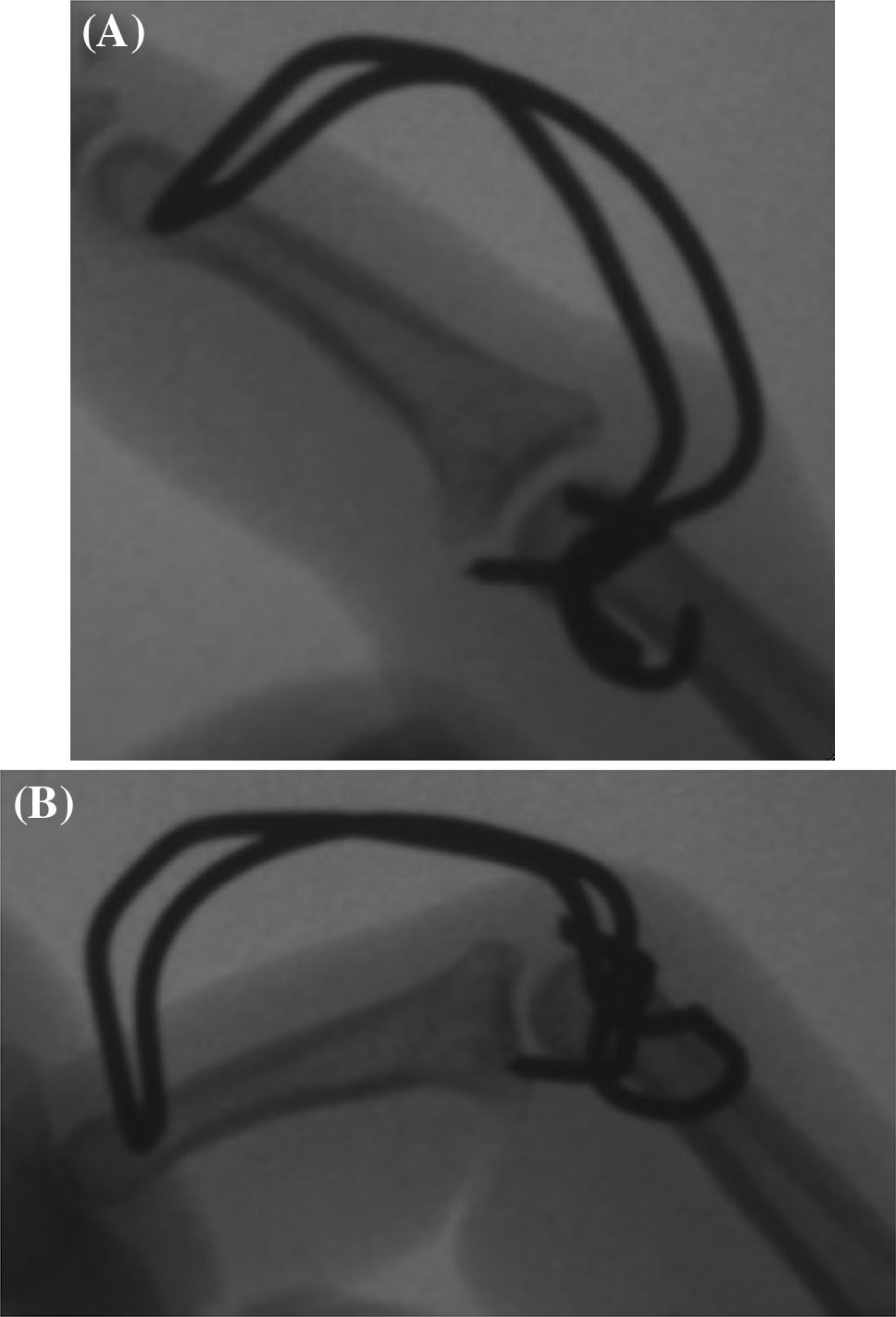
Passive PIP extension (**a**) and flexion (**b**)

After completing the external fixator, congruent passive flexion and extension of the PIP joint were performed under intensifier control in lateral and dorsovolar projections (Fig. 6a, b).

**Fig. 7:**
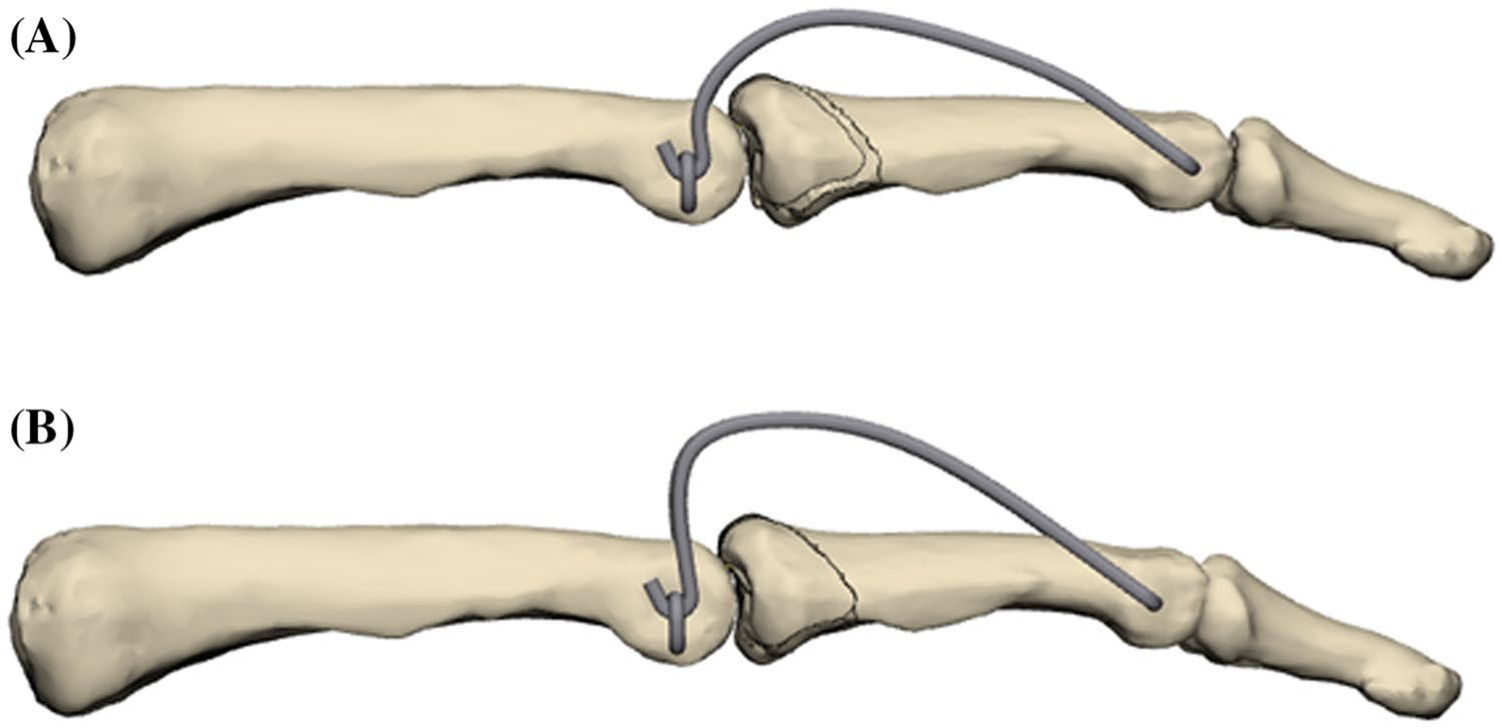
Sagittal parabolic K-wire bending to change distraction strength

If the procedure was done under wide-awake anesthesia, patients were asked to move their finger actively under intensifier control. The remaining subluxation or joint incongruency can be corrected by bending the distal K-wire. A simultaneous change of the radius of both sides of the distal K-wire, into a more or less curved shape in the sagittal plane, allows control of the distraction applied to the PIP joint (Fig. 7a, b).

**Fig. 8:**
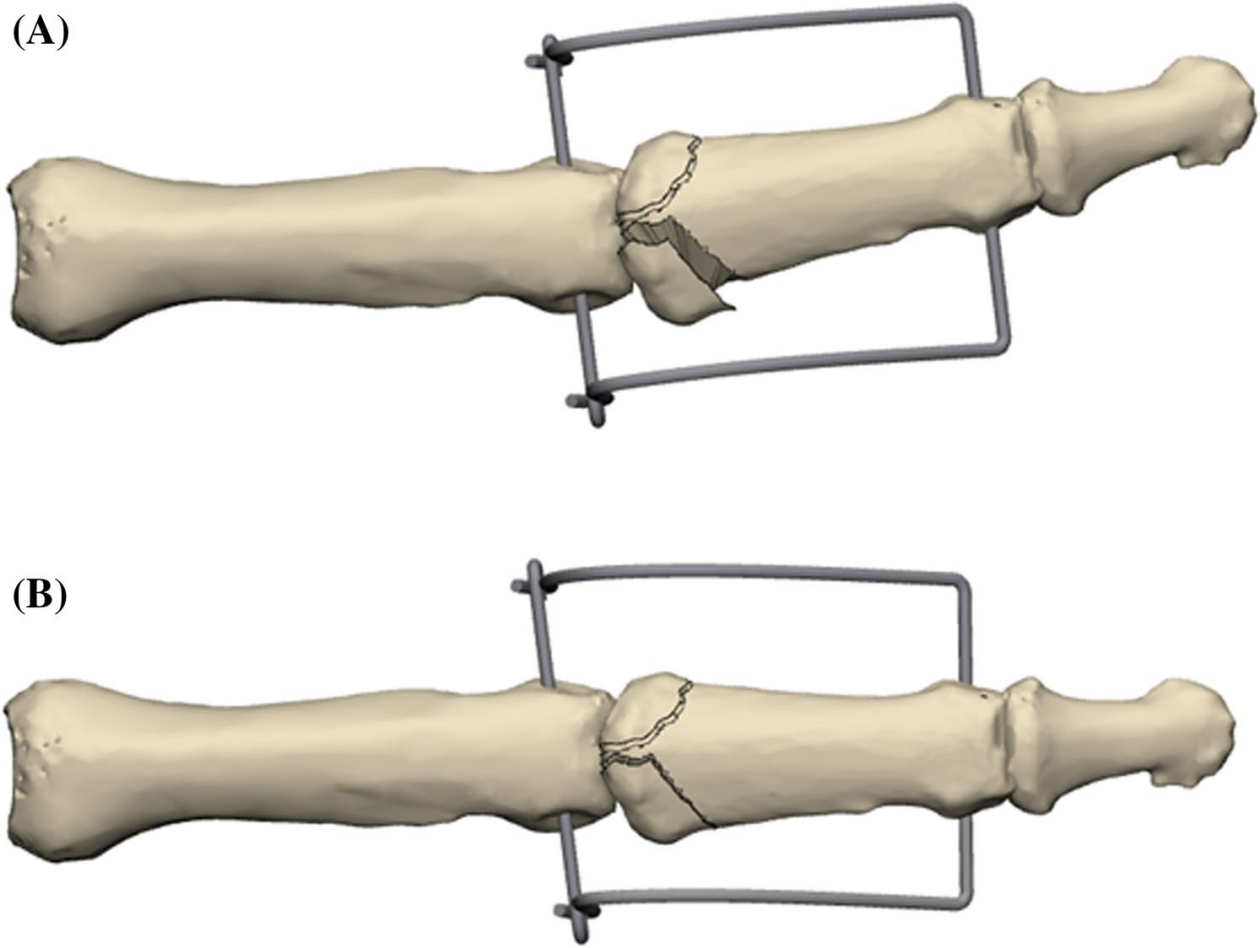
Sagittal parabolic K-wire bending to change distraction strength

Changing the radius of either the radial or ulnar pin in the sagittal plane can correct any radial and/or ulnar deviation (Fig. 8a, b).

### Post-operative rehabilitation protocol

A sterile dressing and a palmar splint were applied intraoperatively. Occupational therapy with guided passive- and active-assisted motion out of the splint was initiated immediately after surgery. The palmar splint was applied until the removal of the fixator 4 weeks postoperatively. The patients were followed-up weekly for pin dressing. No anesthesia was required for the removal of the K-wires.

### Final follow-up assessment

The final follow-up examination included the DASH and the Buck–Gramcko Score questionnaires. Measurement of the active range of motion of the injured and uninjured PIP and the distal interphalangeal (DIP) joints was conducted. Anterior–posterior and lateral X-rays were taken and evaluated for bone healing, signs of osteoarthritis according to the Kellgren–Lawrence Score, remodeling of the joint surface, persistence of any subluxation or fracture displacement, and postoperative deviation of the finger. The patients were asked to rate their pain level using the visual analog scale (VAS, 0 for no pain and 10 for maximum pain), their satisfaction of the treatment on a 1–10 scale (1 most satisfaction–10 worst satisfaction), and their acceptance of the K-wire fixator on a scale from 1–5 (1 most comfort–5 inacceptable).

### Statistical analysis

Statistical evaluation was performed using SPSS version 23 (IBM, Armonk, USA). All descriptive values are expressed as absolute frequencies and percentages or means, standard deviations, and ranges appropriate to data distribution.

Comparisons between the range of motion of the injured joint and the healthy PIP joint of the corresponding finger were performed using the Wilcoxon signed rank test. Statistical significance was set at *p* < 0.05.

## Results

Mean follow-up time was 25.5 months (SD 20.4). The dominant hand was affected fourteen times (66.7%). The index and middle finger were affected once, the ring finger 11, and the small finger 8 times. The frequency of the injury mechanism is shown in Table 1.

**Table 1:** Injury mechanism

Mechanism	Number of patients (*n*)
Downfall	7 (33.3%)
Ball sports	5 (23.8%)
Bike accident	3 (14.3%)
Fight	3 (14.3%)
Skiing accident	2 (9.5%)
Power drill accident	1 (4.8%)

The mean time until surgery was 2.8 days (SD 2.94) after the injury. Ten patients had a palmar displaced fragment which was reduced and fixed with one or two additional 0.8 mm K-wires.

Occupational therapy-guided mobilization of the joint was conducted for an average of 27.1 units (SD 9.8; range 8–42). The external fixator was removed in our outpatient center after a mean period of 35.2 days (SD 6.2 days; range 25–50 days).

### Clinical outcome

The clinical outcome measurements are shown in Table 2. There was a significantly reduced PIP and DIP range of motion in comparison to the contralateral side. PIP joint flexion showed the highest restriction with a mean 30° difference. There was an intermediate acceptance of the fixator (2.7); however, the satisfaction was very high (1.9) and the pain level low (0.7).

**Table 2:** Clinical outcome measurements

	Mean (SD) injured finger	Mean (SD) contralateral uninjured finger	*p* value*
Extension PIP	− 4° (6°)	0° (1°)	0.003
Flexion PIP	80° (22°)	110 (4.75°)	0.000
Range of motion PIP	Absolute 76° Relative^a^ 69%	Absolute 110° Relative 100%	
Extension DIP	− 3° (5°)	4° (1°)	0.000
Flexion DIP	63° (25°)	82° (4°)	0.002
Range of motion DIP	Absolute 60° Relative^a^ 70%	Absolute 86° Relative 100%	
Fingertip volar distance	0.7 cm (2.6 cm)		
Acceptance of fixator (1–5)	2.7 (1.3)		
DASH	11.6 (14.4)		
Buck Gramcko score	13 (3)		
VAS max 1 to 10	0.7 (1.8)		
Satisfaction	1.9 (1.3)		

*p value comparison between the injured and uninjured contralateral finger

^a^Relative range of motion compared to the contralateral uninjured finger

### Radiological outcome

Follow-up radiographs showed bone healing in all cases. At the time of final follow-up, 20 patients (95%) showed a stable aligned and centered joint. Twelve patients achieved boned union in anatomic alignment, whereas seven patients showed a central articular step off of mean 0.3 mm (SD 0.1 mm). One patient showed a deepened articular cavity of 1.2 mm. One patient remained in a subluxated dorsal position and showed a restricted movement with stage 4 osteoarthritis following a pin infection. 85.7% of patients had minimal-to-severe osteoarthritic changes. Three patients showed osteoarthritis stage 0, five stage 1, nine stage 2, three stage 3, and one stage 4.

### Complications

One patient suffered from a recurrent PIP joint dislocation and another from a subluxation of the PIP joint while wearing the fixator. Both joints could be corrected to a centered position by modifying the fixator under image intensifier postoperatively. A third patient sustained a pin tract infection 2 weeks after surgery. As a consequence of this, the external fixator was removed and the fracture was treated with a cast for another 2 weeks.

## Discussion

These results demonstrate that a parabolic dynamic external fixator leads to good clinical and radiologic outcomes in the treatment of pilonoidal PIP joint fracture dislocations.

Promising results are reported using dynamic external fixators by various authors [6, 7, 11–13]. These fixators are advantageous as they allow early motion after surgery avoiding immobilization of the PIP joint, which is the most significant reason for a diminished range of motion [14]. A dynamic external fixator shows beneficial effects on ligamentotaxis realigning the fracture fragments. The applied traction, helped to maintain the disimpacted joint fragment, and the concentric joint position and allowing early joint motion. Moreover, it allows reshaping of the articular surface without compromising the fragile blood supply of the fracture fragments [7]. In our study, twelve patients (57%) showed anatomic alignment, seven a minor step off, one with a deepend articular cavity, and one patient with a subluxed position due to a pin tract infection. All but the latter were stable and showed a concentric joint at the time of final follow-up.

There are several different fixator designs described in the literature [5–8, 13, 15–17]. No major difference in the outcome of the simpler designs compared with the more expensive and sophisticated devices has been described [18]. The present study used a simple and cheap system, only consisting of two K-wires (app. costs < 10 Euro). The main advantage of this design is its simplicity, the limited amount of required hardware, and the possibility for uncomplicated postsurgical readjustments if necessary. An imperfectly placed K-wire can be corrected through changes of the radius of the curvature. Furthermore, there is the possibility to modify the external fixator under image intensifier and without anesthesia if the joint alignment is unfavorable or there is a secondary displacement, which was necessary twice in our study cohort. Both patients healed satisfactorily with a good and pain-free range of motion. The authors faced additional difficulties attempting a secondary modification of the fixator using Suzuki’s technique [19] with K-wires and rubber bands. Fixator movement occurs at the point of contact of the two K-wires and not at the K-wire bone contact point during finger flexion, which is different to other fixator systems. This presumably accounts for a low infection and loosening rate. Numerous reports using external fixators showed a mean range of motion in the PIP joint between 64° and 91° similar to this report with app. 76° [14, 20–22]. The patient cohort in this study showed a moderate acceptance of the external fixator, however, a high satisfaction at final follow-up with very mild discomfort (VAS 0.7). These findings were backed by good functional scores.

**Fig. 9:**
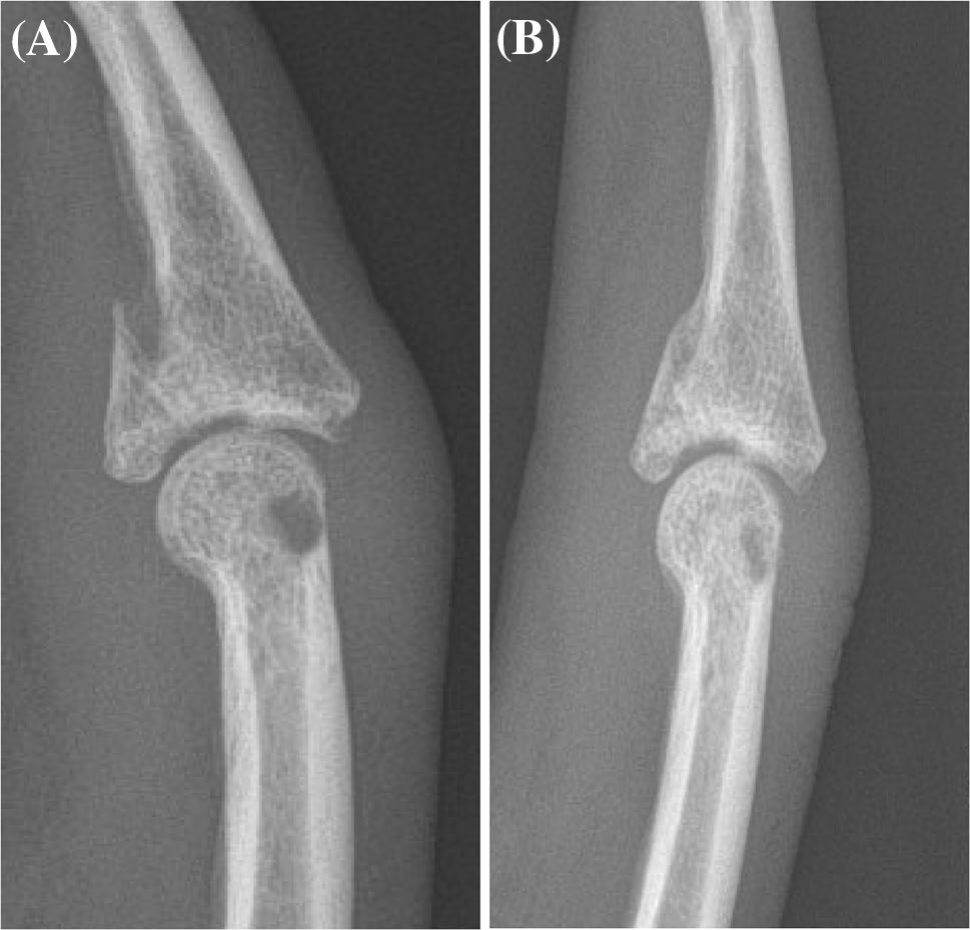
PIP joint after removal of the external fixator; four weeks after surgery with a residual 1 mm articular step off (**a**), and after nine months with a smoothened intraarticular step off (**b**)

Postoperative osteoarthritic changes after treatment with external dynamic fixators develop in 12.5–61% of the cases ranging from mild to moderate changes [7, 8, 23]. In most of the studies, small residual articular step offs are described, which may cause osteoarthritic changes. However, there seems to be no clear association between residual articular step offs and the extent of osteoarthritic changes. Several authors described a short- or long-term remodeling capacity of the injured PIP joint [19, 24, 25]. The present study cohort showed 12 PIP joints anatomically remodeled without any residual intraarticular step off at the time of final follow-up. Seven of the patients still had an articular step off of less than 1 mm. In 14 cases, a residual step off at the time of fixator removal was seen, but was not found on the radiographs at final follow up (Fig. 9a, b). 85.7% of our patients had minimal to severe osteoarthritic changes. However, as the pain level was very low, these osteoarthritic changes did not appear to be symptomatic. This emphasizes the findings of the previous studies which describe an ongoing articular PIP joint remodeling after the removal of the external fixator and no clear correlation to subsequent osteoarthritic changes [19, 24, 25].

The limitations of this study are its retrospective design, the short follow-up period, and the relatively small number of patients, in accordance with other studies. In addition, as this was a cohort study, there was no control group using a different treatment method. As most patients displayed osteoarthritic changes to some extent, long-term follow-up investigations appear necessary to identify patients who show an ongoing increase in osteoarthritis of the PIP joint and possible ongoing reduction of the range of motion.

## Conclusion

The primary results of this report show that the use of a parabolic dynamic external fixator constructed from two K-wires restores joint alignment and stability in unstable pilonoidal PIP joint dislocation fractures. It is a cost-effective alternative to other fixator designs and is associated with a good clinical outcome.
